# A meta-analysis of proactive personality and career success: The mediating effects of task performance and organizational citizenship behavior

**DOI:** 10.3389/fpsyg.2022.979412

**Published:** 2022-10-13

**Authors:** Zeyu Zhang, Han Fang, Yuxiang Luan, Qishu Chen, Jianfeng Peng

**Affiliations:** ^1^School of Labor and Human Resources, Renmin University of China, Beijing, China; ^2^College of Management, Minzu University of China, Beijing, China

**Keywords:** proactive personality, career success, meta-analysis, task performance, OCB

## Abstract

This study aims to reveal the impact of proactive personality on career success (i.e., subjective career success, salary, and promotion) and the sequential mediation effect of organizational citizenship behavior (OCB) and task performance on the relationship. Utilizing meta-analytic structural equation modeling (MASEM) technology sampling 101,131 employees from multiple organizations and industries, which deeply decreased sampling error, the results indicated slightly different findings of proactive personality and three types of career success. Specifically, in relation to salary, OCB and task performance independently transmit the effects of proactive personality to subjective career success, but they sequentially mediate this link as well. In regard to subjective career success and promotion, OCB (but not task performance) mediates the relationship between proactive personality and promotion. OCB and task performance sequentially mediate these links. We discussed findings cautiously and purpose future research directions.

## Introduction

Whether you are an employee or manager in the workplace, a scholar in a business school, or an undergraduate student in a university, you may be interested in the same research topic. This topic is career success (Hirschi et al., 2018). Career success is defined as “the positive psychological or work-related outcomes or achievements one has accumulated as a result of one's work experiences” (Judge et al., 1995, p. 486). Two types of career success (i.e., objective career success and subjective career success) are studied in the career field. Objective career success is measured by using some objective indicators (e.g., salary and promotion, Fuller and Marler, 2009; Spurk et al., 2019) whereas subjective career success is measured by utilizing self-reported scales (e.g., self-reported career satisfaction, Abele and Spurk, 2009; Ng and Feldman, 2014).

Career success is a cumulative outcome produced by the aggregation of behavior over a relatively long period of time (Seibert et al., 1999). Personality may continually influence individuals' behavior and thereby influence their career success. Scholars tried to identify a series of personalities as the antecedents of career success, such as proactive personality (Seibert et al., 1999, 2001; Kim et al., 2009), conscientiousness (Seibert and Kraimer, 2001; Ng and Feldman, 2010), and so on. Early meta-analysis showed that proactive personality has a higher correlation with salary, promotion, and objective career success than conscientiousness (Ng et al., 2005), emphasizing the vital influence of proactive personality on career success. Besides, some longitudinal studies (Seibert et al., 1999, 2001) provided initial causality evidence that proactive personality could predict career success.

Given the important impact of proactive personality on career success, scholars have sought to understand how proactive personality influences individuals' career success. For instance, they found a lot of mediation variables (e.g., innovation Seibert et al., 2001; leader-member exchange, Yang and Chau, 2016; organizational knowledge, Turban et al., 2016) to explain the mechanisms between proactive personality and career success. However, a key mediation variable (i.e., task performance) between proactive personality and career success has not been checked yet. Human resource management informed that employee performance, especially the results of performance appraisal, would influence employees' salary and promotion (Armstrong, 2021). As such, if employees exhibit well performance, they would be recognized by the performance management systems and thereby rewarded by salary and promotion. Proactive personality is found to be positively related to task performance (Fuller and Marler, 2009). That is to say, proactive employees may perform well and thereby achieve career success. Therefore, the first goal of the current study is to reveal the mediating effect of task performance between proactivity personality and career success.

The second purpose of this study is to test the mediating role of organizational citizenship behavior (OCB) in the proactivity–career success linkage. OCB is defined as “individual behavior that is discretionary, not directly or explicitly recognized by the formal reward system, and that in the aggregate promotes the effective functioning of the organization” (Organ, 1988, p. 4). Although OCB would not be directly recognized by the reward system, it may increase individuals' career success. For instance, utilizing the case study method, Grant (2013) found that givers are likely to be successful in the long run. Besides, positive links have been found between OCB and salary (Allen, 2006) and between OCB and salary (Ng et al., 2005). As such, we expect OCB may mediate the link between proactive personality and career success. Besides, from a perspective of social exchange (Ozer, 2011), OCB may influence individuals' task performance. As such, we also examine whether proactive personality will affect career success by OCB and task performance (sequential mediation).

**Figure 1:**
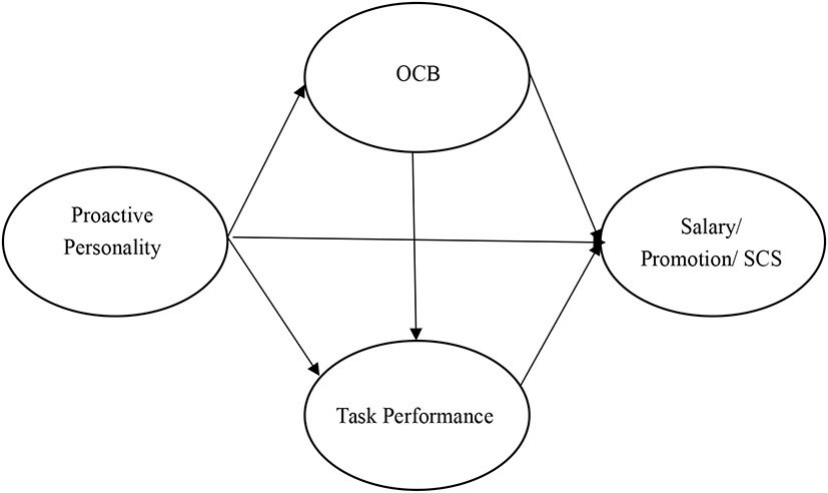
Research model.

Taken together, the purpose of this study is to provide a more complete understanding of the relationship between proactive personality and career success. In particular, in this study, we want to contribute to proactive personality and career success (i.e., subjective career success, salary, and promotion) literature by estimating the mediating (and sequentially mediating) effects of task performance and OCB. We notice that early studies (e.g., Ng et al., 2005; Ng and Feldman, 2010) have accumulated some evidence between proactive personality, task performance, OCB, and career success. But these evidences have not been integrated yet. This study seeks to use meta-analytic structural equation modeling (MASEM) technology to integrate evidence from early primary studies and meta-analyses to test our hypotheses. Compare with traditional SEM, MASEM has at least two advantages. First, when using MASEM, the correlation matrix utilized in the SEM is meta-analytic. As such, researchers could correct some statistical artifacts (e.g., sampling error and measurement error; Hunter and Schmidt, 2004) and thereby get accurate evaluations of interest. Second, MASEM has higher external validity than traditional SEM because data used in MASEM come from different companies and industries (Bergh et al., 2016). This point is especially important in researching the link between personality and career success, because when we sample from only one or two companies, the influence of personality on career success could be inappropriately exaggerated or reduced due to situations. For instance, Judge and Zapata (2015) found personality has a stronger influence on performance in weak situations (e.g., work was unstructured and employees had the discretion to make decisions). That is to say, in a single primary study, the relationships between personality and career success might be influenced by situations. Fortunately, MASEM allows us to eliminate such effect to some extent by synthesizing evidence from different companies and industrials and accurately accomplish our research goals. The overall research model is presented in the Figure 1.

### Theory and hypothesis development

In this part, we will review proactive personality and career success and their measurement. Then, we develop the mediation hypotheses. In an environment that is full of uncertainty, organizations require their employees to accomplish their tasks proactively. That is to say, employees could not always be passive in an uncertain and competitive environment. Scholars note that some individuals are likely to be proactive across situations, indicating that proactivity may serve as a stable trait (Bateman and Crant, 1993). Bateman and Crant (1993) defined proactive personality as individuals' dispositional tendencies to take action to influence their environment. Applying this definition, they developed a 17-items measure of proactive personality. Seibert et al. (1999) developed a short version scale that includes 10 items to measure proactive personality.

Early meta-analyses provided solid evidence for the uniqueness of proactive personality (Thomas et al., 2010; Spitzmuller et al., 2015). Besides, proactive personality has been found positively related to a series of work-related and career-related outcomes, such as work engagement (Bakker et al., 2012), turnover intentions (Akgunduz et al., 2020), career adaptation (Tolentino et al., 2014), career decision self-efficacy (Hsieh and Huang, 2014), and so on, demonstrating the significance of proactive personality in the work and career field.

Compare to proactive personality, career success has a long period of research history. In the labor economy field, some scholars (e.g., Becker, 1962; Mincer, 1970) used human capital theory to explain the antecedents of income since the 1960s. In particular, human capital investment (e.g., education and training) would increase income. Since the 1970s, in the management and vocational psychological field, scholars tried to study the antecedents of career success (Spurk et al., 2019). Four types of variables are usually regarded as the antecedents of career success, namely, human capital (e.g., education level), organizational sponsorship (e.g., supervisor support), socio-demographics (e.g., gender and race), and individual differences (e.g., Conscientiousness) (Ng et al., 2005). Objective career success (i.e., salary and promotion) could be observed by other people directly. However, subjective career success is a subjective rating of one's satisfaction with one's career (Judge and Kammeyer-Mueller, 2007) and could not be observed by other people directly. Subjective career success is usually measured using self-reported scales, such as career satisfaction (e.g., Dyke and Duxbury, 2011; Xie et al., 2016), job satisfaction (e.g., Abele and Spurk, 2009; Cenciotti et al., 2017), and so on.

### The mediating role of task performance

Task performance consists of job-specific behaviors including core job responsibilities (Conway, 1999). For instance, as a salesman, selling products is his/her task performance; as a teacher, teaching student is his/her task performance. Proactive personality should be positively related to task performance. In other words, proactive individuals are likely to demonstrate good task performance. Ability-Motivation-Opportunity (AMO) theory (Appelbaum et al., 2000) helps to explain the link between proactive personality and task performance. This theory argues that performance is determined by ability, motivation, and opportunity. First, proactive individuals are likely to improve their work-related abilities. For instance, proactive individuals are likely to engage in development activities that may relate to their work-related ability (Major et al., 2006). Second, proactive individuals may exhibit a high level of work motivation. For example, early studies found proactive personality is related to two important types of work motivation, namely, work engagement (Bakker et al., 2012; Li et al., 2017) and intrinsic motivation (Joo and Lim, 2009; Karimi et al., 2021). Third, proactive individuals are likely to seek opportunities to achieve task performance. For instance, Crant (1995) suggested that proactive individuals select environments conducive to effective work performance. In other words, proactive individuals are likely to seek opportunities to achieve effective performance by selecting a suitable environment. As such, drawing on a perspective of AMO theory, proactive personality may positively relate to task performance. Besides, a meta-analysis found a moderate and positive correlation (ρ = 0.23) between proactive personality and task performance (Fuller and Marler, 2009).

Task performance should positively link to three types of career success. First, in relation to subjective career success, it should be positively related to task performance. To maintain cognitive consistency or rationalize actions, individuals may adjust their attitudes or cognition to their behavior (Festinger, 1957; Riketta, 2008). As such, when people achieve high performance, they may show satisfaction with their job or career to maintain cognitive consistency. A positive link has been found between task performance and career satisfaction (Cheng et al., 2014). More importantly, longitudinal data supported that job performance could significantly predict subsequent job satisfaction (Blau, 1999). Second, with regard to promotion, it should be positively linked to task performance. Organizations would use performance appraisal as a powerful tool to determine their employees' promotion (Cleveland et al., 1989). Individuals with higher task performance may be observed by performance appraisal and thereby get a promotion. Grabner and Moers (2013) provided evidence that performance could predict promotion decisions. Finally, concerning salary, it should be positively associated with task performance. Under pay-for-performance, the salary is influenced by performance (Meyer, 1975). For instance, the good-performing employees are rewarded with a bonus, whereas the poor-performing employees are rewarded with less bonus or no bonus. A meta-analysis confirmed a positive link between task performance and salary (Ng and Feldman, 2010). Taken together, proactive individuals may exhibit well task performance and thereby achieve three types of career success (i.e., subjective career success, promotion, and salary). Based on the above arguments, we hypothesize:

**Hypothesis 1:** Task performance mediates the relationships between proactive personality and (a) subjective career success, (b) promotion, and (c) salary.

### The mediating role of OCB

Proactive personality should be positively related to OCB. To start, proactive individuals seek to change the environment (Seibert et al., 1999) which is out of their job roles. At the same time, OCB is also a behavior that beyond job requirements (Organ, 1988). Then, proactive individuals would engage in OCB to build a positive social exchange relationship with their organizations and leaders (Li et al., 2010) or to receive social capital (e.g., trust) from the organizations (Yang et al., 2011). Early studies found positive relationships between proactive personality and OCB (Li et al., 2010; Yang et al., 2011).

Organizational citizenship behavior should be linked to career success. First, in relation to subjective career success, it should be positively related to OCB. Helping others is a kind of human nature and would make individuals feel happy (Post, 2005). In the workplace, helping organizations or other employees may trigger satisfaction with the job or career. The experimental-based meta-analysis provided solid evidence for the causality between helping and wellbeing (Curry et al., 2018). As such, when engaging in OCB, employees may feel satisfactory about their job and career. Second, OCB should be linked to promotion. Engaging OCB would help individuals to get a high level of scores in performance evaluation ratings (Whiting et al., 2008) and may thereby help them receive a promotion. Besides, OCB may help employees to build positive social exchange relations with their leaders which could be useful for their promotions. A prior study demonstrated that OCB is positively linked to promotion (Van Scotter et al., 2000). Finally, OCB should be positively associated with salary. OCB may influence leaders' impressions toward their followers and their subsequent behavior. Podsakoff et al. (1993) suggested that managers might consciously recall acts of OCB and intentionally reward the employee out of a desire to reciprocate. Besides, a positive relationship has been found between OCB and salary (Allen, 2006). In sum, proactive individuals may engage in OCB and thereby achieve career success (i.e., subjective career success, promotion, and salary). Therefore, we posit the following:

**Hypothesis 2:** OCB mediates the relationships between proactive personality and (a) subjective career success, (b) promotion, and (c) salary.

### The sequential mediation

When employees help other co-workers, they may build positive social exchange relationships. Subsequently, their co-workers may offer help in return (Ozer, 2011). These helping behavior from co-workers may increase individuals' task performance. Besides, meta-analysis supported the positive relationships between OCB and task performance (Podsakoff et al., 2009). That is to say, OCB may positively link to task performance. As demonstrated earlier, task performance mediates the links between proactive personality and career success (i.e., subjective career success, promotion, and salary). Therefore, OCB triggered by proactive personality may influence task performance, which in turn impacts career success. Taken together, we propose the following hypothesis:

**Hypothesis 3:** The relationships between proactive personality and (a) subjective career success, (b) promotion, and (c) salary are mediated by OCB and task performance (sequential mediation).

## Method

MASEM technology is used to test our hypotheses. Generally speaking, meta-analytic SEM has two steps. The first step is to build a meta-analytic correlation matrix (Viswesvaran and Ones, 1995; Bergh et al., 2016). Such a matrix should include all the relationships we seek to research. These correlations in the matrix could be captured from the early meta-analysis or conducted by an original meta-analysis. The second one is to conduct path analysis applying the meta-analytic correlation matrix we made.

### Building meta-analytic correlation matrix

In this study, following recently-published meta-analyses (Lee et al., 2020; Young et al., 2021), we employed Pearson's *r* was as effect size to represent the strength and direction of the relationship between proactive personality and career success. True score correlation (ρ) was generated utilizing Pearson's *r* after correcting statistical artifacts (e.g., sampling error and measurement error) (Hunter and Schmidt, 2004).

To locate all the potential ρ(*s*) of interest, we searched on the *Web of Science* and *Google Scholar* using the keywords: “proactive personality,” “career success,” “task performance,” “OCB,” “career satisfaction,” and “meta-analysis.” This step helped us to locate the majority of ρ(*s*) which is needed in the SEM analysis. Since the majority of ρ(*s*) provide sufficient information for SEM analysis, we did not further try to search unpublished studies.

**Figure 2:**
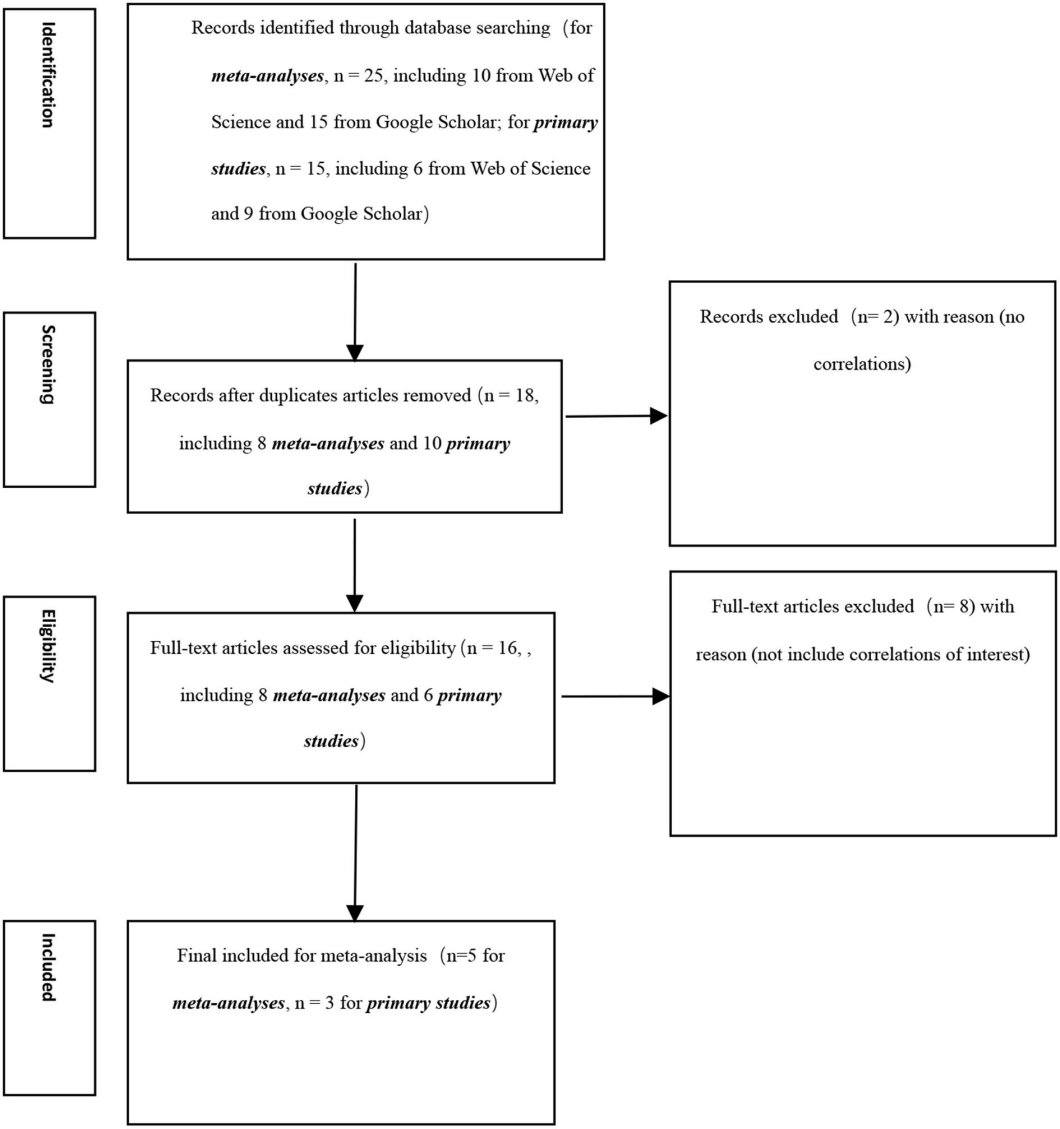
PRISMA flowchart.

Unfortunately, we could not locate a meta-analysis that includes the true score correlation between OCB and promotion. As such, we need to make an original meta-analysis to evaluate their relationships. In particular, we searched for primary studies that include correlations between OCB and promotion. Then we made an original meta-analysis using a random-effect meta-analysis (Hunter and Schmidt, 2004) to evaluate the true score correlation between OCB and promotion. This analysis was accomplished by using the ***psychmeta*** (Dahlke and Wiernik, 2019) package in R. By doing so, we accomplished the full meta-analytic correlation matrix. The correlation required for mediation analysis was presented in Table 1. Besides, a PRISMA flowchart is presented to illustrate the search process (see Figure 2).

**Table 1:** Meta-analytic correlation required for mediation analysis.

**Variable**	***k***	***n***	***r***	**ρ**	***SDρ***	**95% CI**
Proactivity-task performance^1^	8	1,320	0.15	0.23	–	0.14, 0.32
Proactivity-OCB^1^	8	2,116	0.26	0.41	–	0.30, 0.57
Proactivity-salary^1^	10	3,031	0.13	0.14	–	0.05, 0.23
Proactivity-promotion^1^	6	1,737	0.11	0.11	–	0.04, 0.18
Proactivity-subjective career success^1^	32	9,592	0.25	0.31	–	0.27, 0.35
Task performance- subjective career success^2^	217	12,192	0.15	0.17	–	–
Task performance- salary^3^	43	25,220	–	0.19	–	–
Task performance- promotion^3^	34	27,664	–	0.18	–	–
OCB- salary^4^	8	2,631	0.21	0.26	–	–
OCB- promotion^6^	3	935	0.13	0.15	0	0.06, 0.24
OCB- subjective career success^5^	28	6,746	0.24	0.28	0.01	0.26, 0.31
Task performance-ocb^4^	24	7,947	0.39	0.47	0.28	–

k, number of studies; n, total sample size in the meta-analysis; r, uncorrected effect size; ρ, corrected effect size; SDρ, standard deviation of the corrected effect size; CI, confidence interval.

1 Fuller and Marler (2009).

2 Iaffaldano and Muchinsky (1985).

3 Ng and Feldman (2010).

4 Podsakoff et al. (2009).

5 Organ and Ryan (1995).

6 Current study.

### Path analysis

Before analysis, we should explain the cut-off values. In with early published meta-analyses (Ng and Feldman, 2010; Young et al., 2021; Chung et al., 2022; Greco et al., 2022), cut-off values were not considered. This is because the sample in the current study is large enough (*n* = 101,131) so that the cut-off values or outliers would not influence the robustness of the results.

Besides, we introduce the samples in the current study. Generally speaking, the samples used in the current study come from different organizations and industries. This point is very essential when studying personality-performance linkages since the role of personality will be deeply influenced by the environment. As samples come from different organizations and industries, the results could be universal.

**Figure 3:**
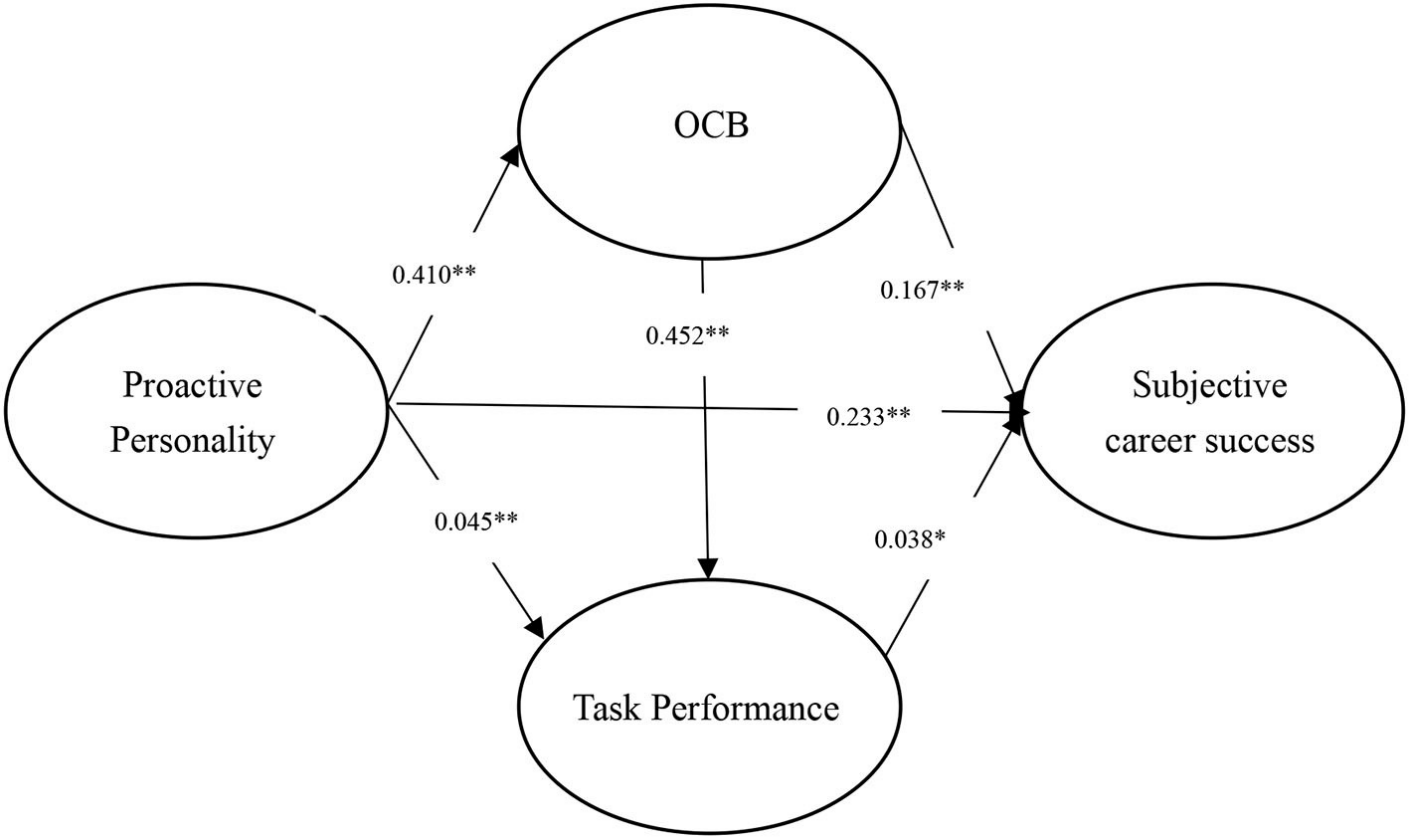
Results of MASEM for proactive personality and subjective career success. **p < 0.01; *p < 0.05.

**Figure 4:**
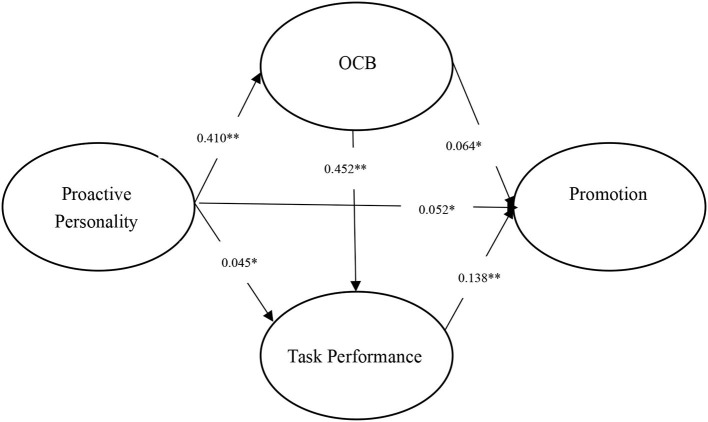
Results of MASEM for proactive personality and promotion. **p < 0.01; *p < 0.05.

**Figure 5:**
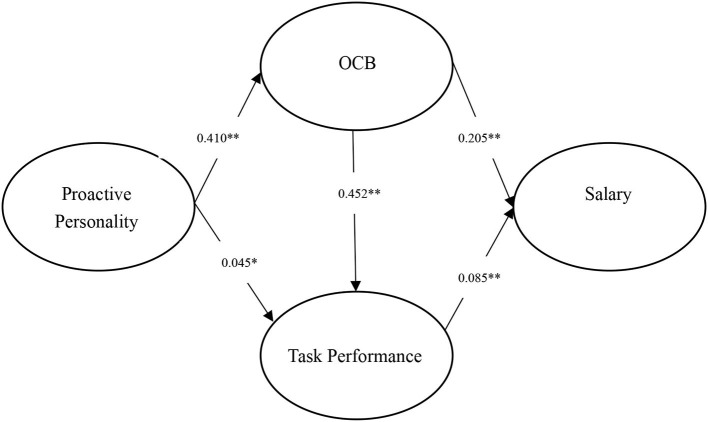
Results of MASEM for proactive personality and salary. **p < 0.01; *p < 0.05.

We conducted path analysis using MPLUS (Muthén and Muthén, 2017) software. In particular, a full correlation matrix was input into MPLUS with a harmonic mean (Viswesvaran and Ones, 1995). Then, we run this software and reported the direct and indirect effects of path analysis. The results are shown in Tables 2–4. We also provided three figures to illustrate the results of path analysis (see Figures 3–5).

**Table 2:** Path coefficient for proactive personality and subjective career success.

**Path**	**Estimate**	**SE**	**95%CI**	***p*-value**
PP → TP	0.045	0.02	0.013, 0.077	0.006
PP → OCB	0.410	0.01	0.383, 0.437	<0.001
PP → SCS	0.233	0.02	0.200, 0.266	<0.001
OCB → TP	0.452	0.02	0.423, 0.481	<0.001
OCB → SCS	0.167	0.02	0.130, 0.203	<0.001
TP → SCS	0.038	0.02	0.003, 0.073	0.032
**Indirect effect**				
PP → TP → SCS	0.002	0.01	0.000, 0.004	0.09
PP → OCB → SCS	0.068	0.01	0.052, 0.084	<0.001
PP → OCB → TP → SCS	0.007	0.01	0.001, 0.014	0.033

PP, proactive personality; TP, task performance; SCS, subjective career success.

N = 3,549 (harmonic mean); CFI = 1; TLI = 1; Chi-Square = 2,021.94; df = 6.

**Table 3:** Path coefficient for proactive personality and promotion.

**Path**	**Estimate**	**SE**	**95%CI**	***p*-value**
PP → TP	0.045	0.02	0.002, 0.087	0.039
PP → OCB	0.410	0.02	0.373, 0.447	<0.001
PP → PR	0.052	0.02	0.005, 0.099	0.031
OCB → TP	0.452	0.02	0.413, 0.490	<0.001
OCB → PR	0.064	0.02	0.012, 0.116	0.017
TP → PR	0.138	0.03	0.089, 0.187	<0.001
**Indirect effect**				
PP → TP → PR	0.006	0.01	0.000, 0.012	0.053
PP → OCB → PR	0.026	0.01	0.005, 0.048	0.017
PP → OCB → TP → PR	0.026	0.01	0.016, 0.035	<0.001

PP, proactive personality; TP, task performance; PP, promotion.

N = 1,975 (harmonic mean); CFI = 1; TLI = 1; Chi-Square = 941.64; df = 6.

**Table 4:** Path coefficient for proactive personality and salary.

**Path**	**Estimate**	**SE**	**95%CI**	***p*-value**
PP → TP	0.045	0.02	0.009, 0.080	0.013
PP → OCB	0.410	0.02	0.379, 0.441	<0.001
PP → SL	0.036	0.02	−0.002, 0.075	0.066
OCB → TP	0.452	0.02	0.419, 0.484	<0.001
OCB → SL	0.205	0.02	0.163, 0.247	<0.001
TP → SL	0.085	0.02	0.045, 0.125	<0.001
**Indirect effect**				
PP → TP → SL	0.004	0.01	0.000, 0.007	0.033
PP → OCB → SL	0.084	0.01	0.065, 0.103	0.017
PP → OCB → TP → SL	0.016	0.01	0.008, 0.023	<0.001

PP, proactive personality; TP, task performance; SL, salary.

N = 2,849 (harmonic mean); CFI = 1; TLI = 1; Chi-Square = 1,462.48; df = 6.

## Results

Three indicators are used to reflect career success. There are subjective career success, promotion, and salary. We will report their results one by one.

### Subject career success

As shown in Table 2 and Figure 3, the indirect effect of proactive personality on subjective career success via task performance (i.e., PP → TP → SCS) is insignificant [β = 0.002, 95% CI = (0.000, 0.004), *p* = 0.09 > 0.05]. As such, H1 (a) was rejected. Then, the indirect effect of proactive personality on subjective career success *via* OCB (i.e., PP → OCB → SCS) is positive and significant [β = 0.068, 95% CI = (0.052, 0.084), *p* = < 0.001]. Therefore, H2 (a) was accepeted. Finally, the indirect effect (i.e., PP → OCB → TP → SCS) of proactive personality on subjective career successs through OCB and task performance (sequential mediation) is significant [β = 0.007, 95% CI = (0.001, 0.014), *p* = 0.033 < 0.050]. Thus, H3 (a) was accepeted. To sum up, results reveal that proactive personality and subjective career success are not linked through task performance, but through (a) OCB and (b) OCB and then task performance.

### Promotion

As presented in Table 3 and Figure 4, we notice that the indirect effect of proactive personality on promotion *via* task performance (i.e., PP → TP → PR) is insignificant [β = 0.006, 95% CI = (0.000, 0.012), *p* = 0.053 > 0.050], rejecting H1(b). Then, the indirect effect of proactive personality on promotion *via* OCB (i.e., PP → OCB → PR) is positive and significant [β = 0.026, 95% CI = (0.005, 0.048), *p* = 0.017 < 0.050], supporting H2 (b). Finally, the indirect effect (i.e., PP → OCB → TP → PR) of proactive personality on promotion through OCB and task performance (sequential mediation) is significant [β = 0.026, 95% CI = (0.016, 0.035), *p* = < 0.001], confirming H3 (b). Together, findings suggest that proactive personality and promotion are not linked through task performance, but through (a) OCB and (b) OCB and then task performance.

### Salary

As provided in Table 4 and Figure 5, we found that the indirect effect of proactive personality on salary *via* task performance (i.e., PP → TP → SL) is significant [β = 0.004, 95% CI = (0.000, 0.007), *p* = 0.033 < 0.050], supporting H1(c). Then, the indirect effect of proactive personality on salary *via* OCB (i.e., PP → OCB → SL) is positive and significant [β = 0.084, 95% CI = (0.065, 0.103), *p* = 0.017 < 0.050], supporting H2 (c). Finally, the indirect effect (i.e., PP → OCB → TP → SL) of proactive personality on salary through OCB and task performance (sequential mediation) is significant [β = 0.016, 95% CI = (0.008, 0.023), *p* = < 0.001], confirming H3 (c). Overall, results demonstrate that proactive personality and salary are linked through (a) task performance, (b) OCB, and (c) OCB and then task performance.

## Discussion

In this study, we seek to answer *why* proactive individuals tend to achieve career success by investing in the mediating effects of task performance and OCB. As our path analysis is based on meta-analytic evidence, the results are more reliable than a single primary study. More importantly, our data come from different industries and companies, helping us to eliminate the influence of sampling. This point is very important in researching the influence of personality on career success because the effects of personality may vary due to the strength of situations (e.g., industries and companies) (Judge and Zapata, 2015). Overall, we found that task performance and OCB mediate the link between proactive personality and career success. However, minor differences have been found when using three indicators to operationalize career success. We will discuss them. Management implications, limitations, and future research directions are discussed.

### The mediating role of task performance

This study contributes to proactive personality and career success literature by revealing the mediating effect of task performance. In H1, we hypothesized that task performance will mediate the links between proactive personality and three types of career success. However, this hypothesis was accepted partially. In particular, this mediating effect is significant only when career success is operationalized as salary. Judge and Kammeyer-Mueller (2007) suggested that task performance may mediate the link between personality and career success. Our study provides evidence for Judge and Kammeyer-Mueller (2007)'s argument. Interestingly, when career outcome is salary rather than subjective career success and promotion, the results are significant. A plausible explanation is that task performance is more likely to influence salary straightforwardly as performance appraisal would influence salary directly. However, for subjective career success and promotion, the effect of task performance may be more weak and indirect.

### The mediating role of OCB

This study contributes to OCB literature by revealing the important role OCB plays in explaining the personality-career success linkage. In H2, we hypothesized that OCB will mediate the links between proactive personality and three types of career success. This hypothesis is fully accepted. Our study provides an OCB mechanism for understanding the role of proactive personality on career success. Engaging in OCB may cost employees time and energy. For instance, Koopman et al. (2016) found engaging OCB interferes with perceptions of work goal progress. However, career success is a long-term product (Judge et al., 1995). In the short term, proactive employees' helping behavior may consume their own time and energy. However, in the long run, as our data demonstrated, the benefits of this behavior may outweigh its costs and thereby bring career success. This is in line with the argument by Grant (2013). In his book *Give and Take*, he suggested that givers are likely to be successful in the long run.

### The sequential mediation

Our finding also supported the sequential mediation effect of OCB and then task performance in three types of career success. This finding contributes to our knowledge of the deeply potential mechanisms behind the proactive personality-career success linkage. Although time spent on OCB may decrease the time spends on task performance (Bergeron et al., 2011), our results support the positive role of OCB on career success through task performance. Our study also responded to Seibert et al. (1999)'s suggestion that future studies should detect the behavior mediators between proactive personality and career success.

### Management implications

Our research highlights several important management implications. First, we reveal the mechanism between proactive personality and career success. This finding helps the human resource management department to understand why proactive employees tended to be successful, helping them to make employees' career development plans. In particular, the human resource management department could try to influence employees' cognition of proactive personality and take more proactive behavior (Zhou et al., 2021). Second, as we found task performance is crucial to understanding the relationship between proactive personality and career success, organizations should improve their performance management systems. Specifically, performance appraisal systems should be improved to capture task performance accurately and provide feedback to proactive employees. Finally, the result shows the important role of OCB in career success. Organizations could try to build a climate in which OCB is encouraged as such proactive individuals may engage in OCB and thereby achieve career success. This point is very vital in today's environment because performance is dependent on the cooperation of team members and OCB is crucial for team members' cooperation (Beersma et al., 2003).

### Limitations are future directions

Some limitations should be mentioned. First, as we employ ρ as effect size in our meta-analysis, we could not get the accurate causation between variables. For instance, employees who have a high-level salary or feel satisfied with their career may also have the motivation to engage in OCB. Future studies could use experimental research design to conclude accurate causation. Second, both subjective career success and proactive personality are usually measured by self-reported scales. That is to say, the relationships may suffer from common method bias (Podsakoff et al., 2003). Future studies could try to use a time-lagged research design to decrease the influence of common method bias. Finally, as we collected correlations from well-established meta-analyses, we could not conduct a publication bias analysis. As such, the potential influence of unpublished studies remains unclear. Future study could try to make an original meta-analysis that included unpublished studies to check the potential publication bias.

## Conclusions

Scholars paid so much attention to revealing the link between personality and career success.

This study seeks to answer why proactive individuals tend to achieve career success. We apply meta-analytic SEM to reveal the mediating effect of task performance and OCB on the proactive personality-career success linkage. OCB is regarded as an important mediator to explain the relationship between personality and career success for three indicators of career success (i.e., subjective career success, salary, and promotion). However, task performance only mediates the link between career success and salary. The relationship between proactive personality and career success is mediated by OCB and task performance (sequential mediation). Our study contributes to proactive personality, OCB, task performance, and career success literature. Our study also provides insights into human resource management as employees' career development is very vital for organizations. Hoping our study will raise scholars' and managers' continuous interest in personality and career success.

## Data availability statement

The original contributions presented in the study are included in the article/supplementary material, further inquiries can be directed to the corresponding author.

## Author contributions

ZZ and JP: ideas. ZZ, HF, and YL: introduction to method. QC: discussion to results. All authors contributed to the article and approved the submitted version.

## Conflict of interest

The authors declare that the research was conducted in the absence of any commercial or financial relationships that could be construed as a potential conflict of interest.

## Publisher's note

All claims expressed in this article are solely those of the authors and do not necessarily represent those of their affiliated organizations, or those of the publisher, the editors and the reviewers. Any product that may be evaluated in this article, or claim that may be made by its manufacturer, is not guaranteed or endorsed by the publisher.
